# Dynamic force microscopy simulator (dForce): A tool for planning and understanding tapping and bimodal AFM experiments

**DOI:** 10.3762/bjnano.6.36

**Published:** 2015-02-04

**Authors:** Horacio V Guzman, Pablo D Garcia, Ricardo Garcia

**Affiliations:** 1 Instituto de Ciencia de Materiales de Madrid, CSIC, Sor Juan Inés de la Cruz 3, 28049 Madrid, Spain

**Keywords:** bimodal AFM, dynamic AFM, nanomechanics, numerical simulations, tapping mode AFM

## Abstract

We present a simulation environment, dForce, which can be used for a better understanding of dynamic force microscopy experiments. The simulator presents the cantilever–tip dynamics for two dynamic AFM methods, tapping mode AFM and bimodal AFM. It can be applied for a wide variety of experimental situations in air or liquid. The code provides all the variables and parameters relevant in those modes, for example, the instantaneous deflection and tip–surface force, velocity, virial, dissipated energy, sample deformation and peak force as a function of time or distance. The simulator includes a variety of interactions and contact mechanics models to describe AFM experiments including: van der Waals, Hertz, DMT, JKR, bottom effect cone correction, linear viscoelastic forces or the standard linear solid viscoelastic model. We have compared two numerical integration methods to select the one that offers optimal accuracy and speed. The graphical user interface has been designed to facilitate the navigation of non-experts in simulations. Finally, the accuracy of dForce has been tested against numerical simulations performed during the last 18 years.

## Introduction

Numerical simulations have played a pivotal role to advance the understanding and, in the process, to improve the performance of amplitude modulation atomic force microscopy (AM-AFM), usually known as tapping mode AFM. The following discussion provides some examples. Simulations provided the first estimation of the forces and deformations involved in tapping mode AFM [1–2]. They explained the origin of the phase contrast observed on heterogeneous samples by tapping mode AFM in air [3] and liquid [4–5]. In the process, simulations validated the theory of AFM phase imaging in air [6–7], its use to identify energy dissipation processes [7] or to measure the energy dissipated in the sample [8–10]. Numerical simulations have provided critical insight to understand the subtle nonlinear dynamics aspects present in AM-AFM, such as the existence of multiple interaction regimes [11–13] or the presence of chaotic tip motion [14]. Similarly, simulations have linked the presence of higher harmonic components in the tip motion with the presence of nonlinear interactions [15]. In the process, the cross talk between modes and harmonics has been clarified [16–20]. The complicated cantilever motion in liquid and the differences observed between the excitation methods have been analyzed by simulations [21–23].

The tip–surface force controls the cantilever motion, however, the force itself is not an observable. Numerical simulations have been used to derive parametric approximations [24], scaling laws [25] and insights about the role of different material properties [25–27] in obtaining the maximum force. Simulations can generate maps that provide the estimation of the peak forces for a large variety of conditions [27–28]. The range of applicability of the force reconstruction methods has also been verified by numerical simulations [29]. The spatial resolution and contrast of different dynamic AFM methods has also been studied by simulations [28,30–31]. Finally, the emergence of multifrequency AFM [32] in particular bimodal [33–34], trimodal [35], intermodulation [36] or torsional harmonics [37] has been supported by simulations [38]. In the case of bimodal AFM, numerical simulations [39] preceded and paved the way to its experimental development [33,40].

The complexity of amplitude modulation AFM makes it difficult to develop reliable code accessible for both the large community of tapping mode AFM users and the emerging community of multifrequency AFM. The future applications and understanding of dynamic AFM operation will be enhanced if accurate simulators are easily accessible to the experimentalist. These factors promote the development of AFM simulation platforms such as VEDA [41–42].

Here we present a dynamic AFM simulator (dForce), which is based on the experience and knowledge accumulated from nearly 20 years of simulations. The interactive simulator has a modular structure that allows AFM users from the unexperienced to the most advanced to simulate a wide variety of experimental conditions and/or operational modes. The code is valid for air or liquid environments, soft or hard materials, small or large amplitudes, conservative and non-conservative forces and single or bimodal excitation modes. Its accuracy has been tested against previous numerical simulations. The dForce simulator will be useful to either devise the optimal experimental conditions in terms of amplitudes, peak forces, material property sensitivity and spatial resolution, or to explain the experimental data in standard and non-standard dynamic AFM configurations.

**Figure 1:**
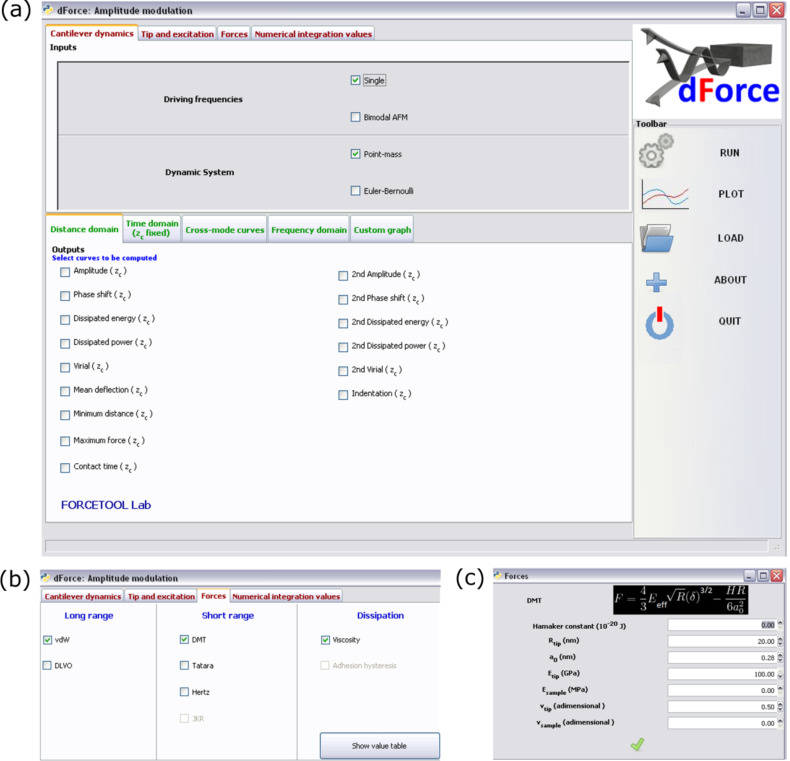
Graphical user interface of dForce. (a) Main menu. It is divided in three sections, two horizontal and one vertical. The top tabs contain all panels relevant for AFM simulations. The bottom panel enables selection of the output plots. The vertical panel contains the tabs for running the program and managing the output. (b) Force panel that shows the different tip–surface force models implemented in dForce. (c) Example of the input panel for the DMT model.

The code is written in Python/SciPy, which is embedded with open source features. It can be run on Windows, Mac and Linux operating systems. It can be freely downloaded from our website [43]. The user interface has been designed to mimick some of the main steps of an amplitude modulation AFM experiment (Figure 1).

The dForce user must be aware that the accuracy of the simulations to describe an experiment cannot be better than the accuracy of the model used to describe it. In addition, the use of dForce should be accompanied by an understanding of the physics of dynamic AFM methods. There are many instances where a poor selection of the different parameters in the code could generate incorrect results without producing errors in either the model or the code.

## Results and Discussion

### Cantilever–tip dynamics

In amplitude modulation AFM the equation of motion for the microcantilever–tip system is approximated by using the point-mass model [11],

[1]
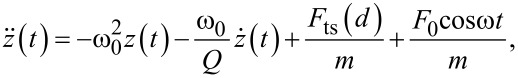


where *m* is the effective mass of the cantilever tip, ω_0_ is the angular resonant frequency, *Q* the quality factor, *k* the spring constant of the fundamental resonance (first flexural mode) and *F*_ts_ is the tip–sample interaction force. The above equation is applicable when the contributions from higher modes to the cantilever motion are negligible [15].

The presence of higher flexural modes in the the cantilever–tip motion can be described by using a continuos beam theory [15–16]. The extended Euler–Bernoulli equation considers the cantilever as a continuous and uniform rectangular beam under the action of external forces,

[2]
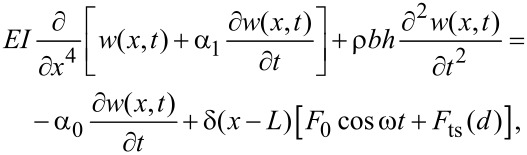


where *E* is the Young modulus of the cantilever, *I* the area moment of inertia, α_1_ the internal damping coefficient of the cantilever, ρ the cantilever mass density, *b*, *h* and *L* are the width, thickness and length of the cantilever, respectively, α_0_ is the hydrodynamic damping of the medium, and *w*(*x*,*t*) is the time-dependent, vertical displacement of the differential beam’s element placed at the *x* position. To numerically solve the above equation, we replace it by a system of point-mass equations, one for each relevant mode, *n* = 1, 2, etc. as described by [16,44–45]

[3]
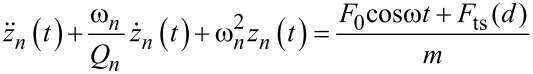


with *m* = 0.25·*m*_c_ and 1 + cos (κ*_n_*) cosh (κ*_n_*) = 0, where κ*_n_* is the *n*th positive real root of the above equation and *m*_c_ is the real mass of the cantilever. Additionally, the quality factor is defined as

[4]
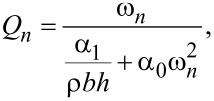


where

[5]
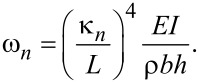


### Tip–sample interaction forces

The simulator includes a variety of models and tip–surface force interactions. The interactions are separated in long range and short range. The user has the option of combining a long range with a short range interaction to produce the full tip–surface force. It is also possible to add one or several non-conservative interactions such as adhesion hysteresis and/or viscoelasticity. We briefly describe the resulting expressions.

#### van der Waals

The van der Waals interaction between a sphere and a half-space is calculated by [46]

[6]
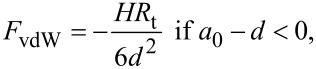


where *R*_t_ is the tip radius, *H* is the Hamaker constant, *d* is the distance between the tip’s apex the sample surface and *a*_0_ is the intermolecular distance (0.165 nm).

#### Derjaguin–Landau–Verbey–Overbeek (DLVO)

The DLVO force [46] describes interactions in liquid by including the contributions from the electrical double layer and the van der Waals interactions. The DLVO force is given by

[7]
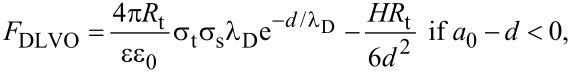


where λ_D_ is the Debye length, ε is the relative permeability, ε_0_ is the vacuum permeability, σ_t_ is the tip–surface charge density and σ_s_ is the sample surface charge density.

#### Hertz contact mechanics

The elastic contact between the tip and sample is usually modelled with the Hertz model [46] whereby for a spherical tip and a half-space sample the force is given by

[8]
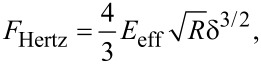


where δ is the indentation and *E*_eff_ is the effective Young modulus of the interface defined by

[9]
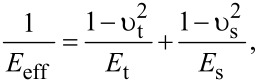


where *E*_t_ and *E*_s_ are the Young’s modulus of the tip and sample, respectively, and υ_t_ and υ_s_ are the Poisson coefficients of the tip and sample, respectively.

#### Derjaguin–Mueller–Toporov contact mechanics (DMT)

The DMT model is valid for describing stiff and small contacts with low adhesion forces. The DMT model [47] considers an elastic term given by Hertz contact mechanics as

[10]
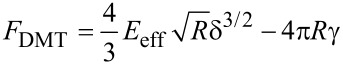


and an adhesion force that acts outside the contact area given by

[11]
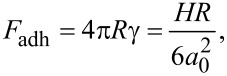


where γ is the sample surface energy.

#### Johnson–Kendall–Roberts contact mechanics (JKR)

The JKR model is applied to describe contacts characterized by a relatively small Young modulus, and large adhesion and contact area [48]. In this model the force is calculated as an implicit equation of the indentation

[12]
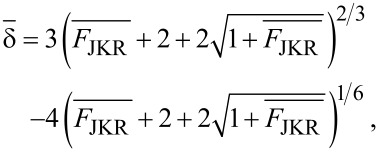


where

[13]
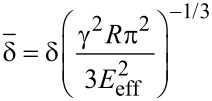


and

[14]
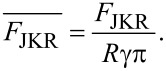


#### Tatara contact mechanics

The Tatara contact mechanics are applied to describe a sample with a finite size with respect to the tip. It releases the vertical load into both vertical and lateral deformations [49]. The deformation is symmetrically generated on both sides of the sample, one that is in contact with the tip and the one in contact with the substrate. The force is calculated by

[15]
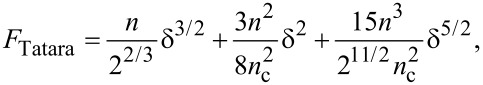


with

[16]
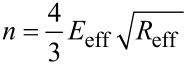


[17]
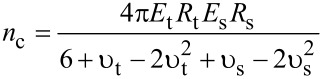


[18]
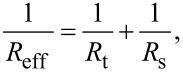


where *R*_t_ and *R*_s_ are the tip and the sample radius, respectively.

#### Bottom effect cone correction (BECC)

This model was recently introduced by Gavara and Chadwick to suppress the influence of the stiffness of the substrate on the stiffness measured by AFM on very soft and thin materials deposited on them [50]. The expression is valid when the Young modulus of the substrate is several orders of magnitude higher than that of the sample. The force is calculated by

[19]
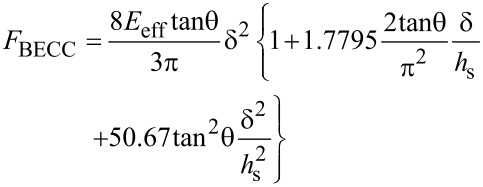


where θ is the half-cone angle and *h*_s_ is the thickness of the sample.

#### Linear viscosity force

The linear viscosity force deduced by Garcia and San Paulo [51] combines the relationship between the stress and strain given by the Kelvin–Voigt model and the sample deformation given by Hertz contact mechanics as

[20]
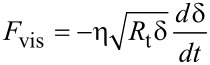


where η is the viscosity coefficient.

#### Standard linear solid viscoelastic model (SLS)

The SLS model is considered to represent the time-dependent behavior of a viscoelastic material without residual strains [52]. The model characterizes a viscoelastic material as an elastic element, which is coupled in series with a system that includes another elastic element and a viscous response. The equivalent mechanical system is a spring in series with a spring and a dashpot. By assuming a contact mechanism as described by Hertz contact mechanics, we deduce the force as

[21]
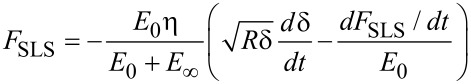


where *E*_0_ and *E*_∞_ represent the Young’s modulus of the material at fast and slow loading rates, respectively.

#### Customized force

The code also enables the definition of other types of forces. For that purpose, the advanced user could use any of the variables and/or parameters defined in the above force models, or could use any or several of the four undefined parameters *P*1, *P*2, *P*3 and *P*4 allowed by the code. In this manner, the code has the capability to simulate the dynamics of the microscope under a force that includes any of the variables of the above models and up to four new parameters generally defined as

[22]
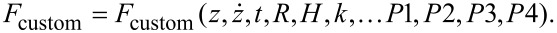


#### Numerical integration methods

To obtain the cantilever–tip motion in AM-AFM we have considered a point-mass model with the parameters of the first flexural model of the cantilever. For bimodal AM we have considered a system of equations involving the first three flexural modes [39,45]. Each mode was described by a point-mass model. In this system the modes are coupled by the tip–sample interaction force. The equations of motion were integrated numerically using the Livermore solver for ordinary differential equations (LSODA) of the scipy library (scipy.ode) [53–55]. LSODA uses an algorithm to adapt the integration step for a given numerical tolerance of the numerical integration [51]. We have chosen this numerical method because it offers faster integration times as compared to the commonly used fourth-order Runge–Kutta algorith (RK-4) [56]. We have compared the numerical integration for AM-AFM and bimodal AM microscopy examples (see Table 1). The results presented in Table 2 show the performace of LSODA with respect to RK-4. In all the cases, LSODA performs the simulations significantly faster than RK-4. In fact, when higher accuracy is demanded (higher *m* values), LSODA also performs better. We note that the comparison applies only to the part of the code that involves the integration methods and not to the code as a whole, which involves other operations such as data storing and visualization.

**Table 1:** Parameters used to compare the performance of the numerical integration algorithms LSODA and Runge–Kutta 4 (RK-4) for *z*_c_ = 4 nm.

AFM configuration	*R* (nm)	*f*_1_ (kHz)	*k*_1_ (N/m)	*Q*_1_	*A*_01_ (nm)	*f*_2_ (kHz)	*k*_2_ (N/m)	*Q*_2_	*A*_02_ (nm)	*E*_s_ (Pa)	*H* (J)

AM-AFM	2	48.9	0.9	100	5	–	–	–	–	1 G	0
Bimodal AM	20	48.9	0.9	100	5	282	28	200	1	1 G	0

**Table 2:** Comparison between two different numerical integration algorithms, LSODA and RK-4. The numbers indicate the factor by which LSODA is faster than RK-4 for two dynamic AFM configurations (see Table 1); *m* is the number of points per period to represent the oscillation.

	*m* = 128	*m* = 256	*m* = 512

AM-AFM	2.8	4.3	6.5
Bimodal AM	4.5	7.3	11.1

For the simulations, it is important to choose appropriate values for the numerical integrator, in particular the number of periods, interval of periods (to calculate the steady state), and the average tip–surface distance step to guarantee that the simulations reflect the proper oscillation conditions. In general, the main interest is in the steady state solutions, that is, when the transient term has practically vanished [46]. For a driven and damped harmonic oscillator, the transient terms are reduced by a factor of 1/e after a time *t* = *QT*/π where *T* is the natural period of the oscillation. Consequently, the number of periods should be at least 2–5 times larger than *Q*/π for quality factors above 30. For a smaller *Q* values, it should involve about 30 periods. The interval of periods to calculate the steady state solution refers to the number periods in the oscillation that will be used in the calculations. These are the last periods of the total number of periods and typically 8 periods are sufficient. The last key parameter is the tip step. This refers to the amplitude of the motion of the tip (phase shift) versus distance curves. Smaller steps will give better results but they imply larger computation times. A 1 nm *z*_c_ step is a good starting value, however, smaller values could be necessary depending, for example, on the coexistence of attractive and repulsive interaction regimes.

#### Graphical user interface

The graphical user interface is divided into three main sections: microscope input data, the output section and the toolbar for running the program and handling files. Figure 1a shows a screenshot of the interface. In the AFM data panel some tabs facilitate the introduction of the relevant information for the simulation such as the type of AFM configuration (AM-AFM versus bimodal AFM) or the model to describe the tip–surface forces. By activating any of the tabs a new panel will show the available options. Figure 1b shows the options available to model the tip–surface interactions. An example of force data panel is shown in Figure 1c.

#### Amplitude modulation AFM simulations

The results presented in this section describe steady-state conditions. Consequently, the numerical integration values have been adjusted to integrate the tip’s deflection once the transient component has faded away [57]. The parameters characterizing the AFM operation used in the Figures of this section are summarized in Table 3.

**Table 3:** Summary of the parameters used in the numerical simulations described in Figures 2–5.

Figure	*R* (nm)	*f*_1_ (kHz)	*k*_1_ (N/m)	*Q*_1_	*A*_0_ (nm)	*E*_s_ (Pa)	*H* (J)	η (Pa·s)

2	2	49.9	0.9	100	1	1 G	0.8 × 10^−20^	0
3 (air) (water)	2	48.9	0.9	255 2	10	1 G	2 × 10^−20^ 0	0 0
4	2	48.9	10	100	10	50 G (hard) 50 M (soft)	0	0
5	2	49	0.9	50	10	50 M	0	100

**Figure 2:**
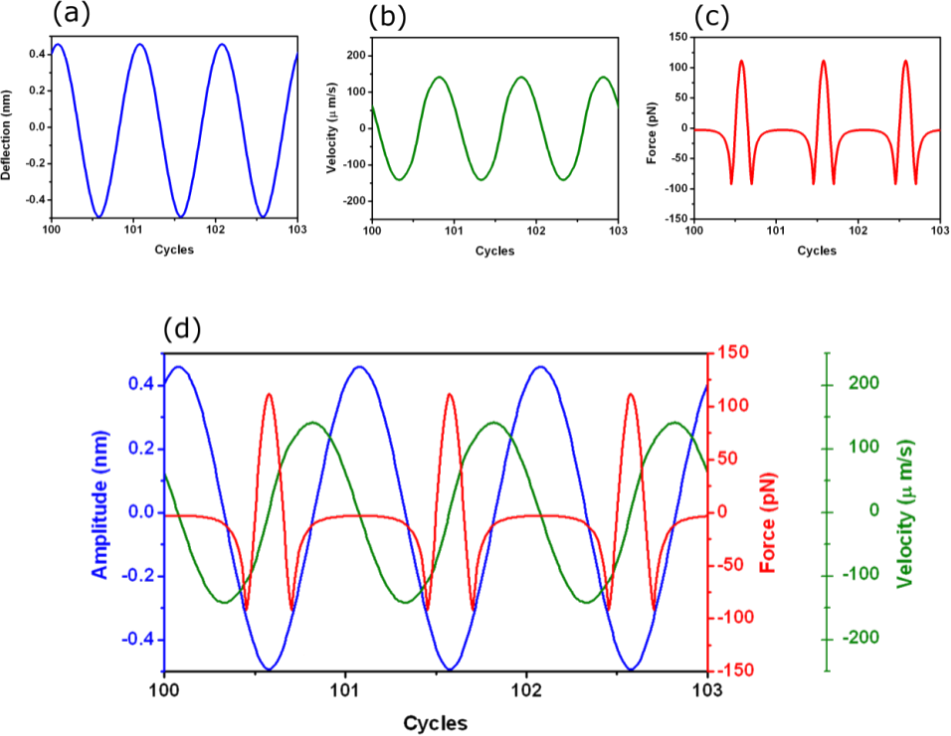
dForce simulation of AM-AFM for a tip–surface force that includes van der Waals and DMT. (a) Instantaneous tip deflection. (b) Instantaneous velocity. (c) Instantaneous force. (d) Combining different plots in a single figure. Deflection (blue), velocity (green) and force (red). The simulations were performed for *z*_c_ = 0.5 nm.

Figure 2 shows the tip motion under the influence of a force that includes van der Waals and Hertz contact mechanics (see Table 3 for the parameters). The instantaneous tip deflection, velocity and force are shown. The tip oscillates between ±0.47 nm while the velocity changes between zero and 141 μm/s. The instantaneous force shows the presence of a non-interacting region for distances larger than the set point amplitude, the region of the attractive force and the region of repulsive forces. In this case, attractive and repulsive peak forces are about −92 and 112 pN, respectively. One useful feature provided by dForce is the capability to combine different data in a single plot. Figure 2d shows the cantilever deflection, the velocity and the force.

#### Coexistence of interaction regimes

**Figure 3:**
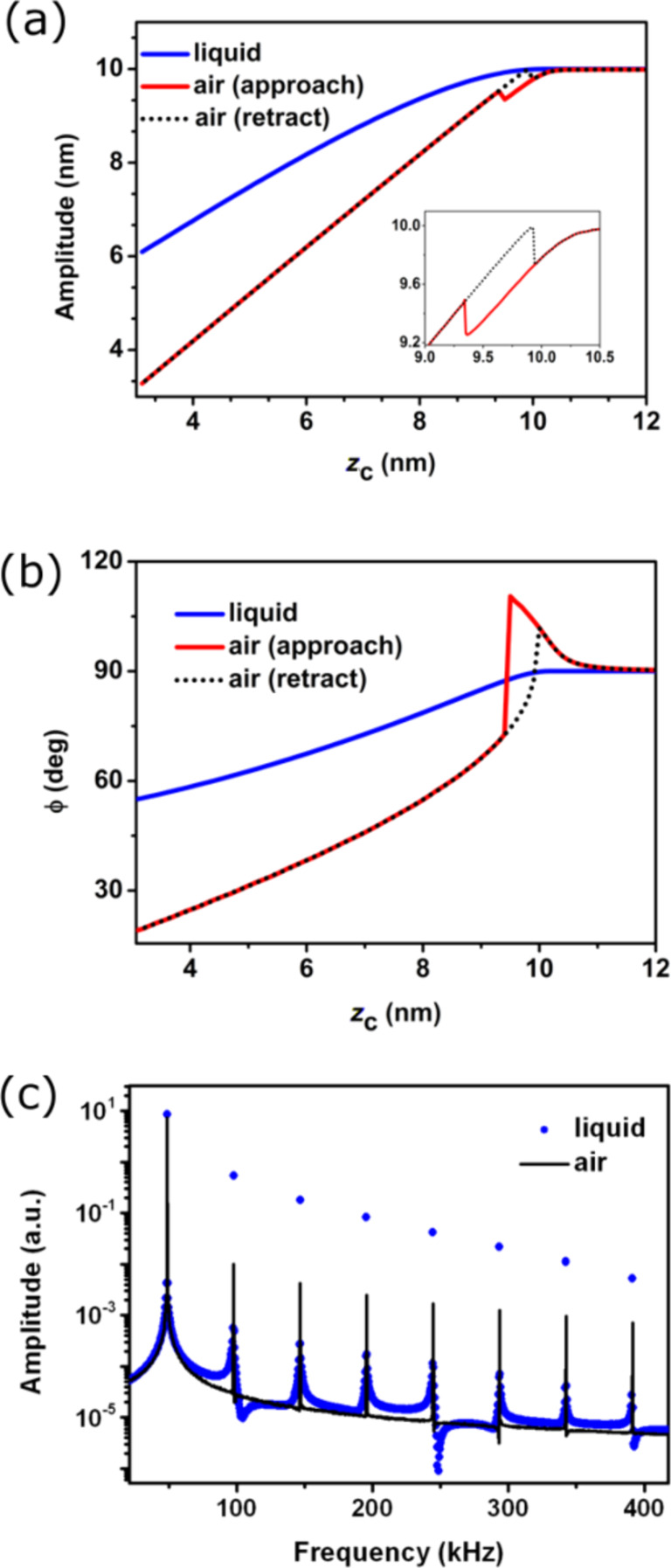
AM-AFM comparison of cantilever dynamics, air versus liquid. (a) Amplitude versus average tip–surface distance curves for low and high Q values. The inset shows the details of the hysteresis in the amplitude (approaching and retraction cycles). (b) Corresponding phase shift curves. (c) Spectra of the amplitude components taken at *z*_c_ = 7 nm. The approaching (continuous line) and retraction curves (dots) are also shown in (a) and (b) for *Q* = 255.

The existence of different interaction regimes in AM-AFM is a direct consequence of the nonlinear character of the tip–surface force [11,46]. The amplitude versus the average tip–surface distance *z*_c_ curve shows a sudden increase at *z*_c_ = 9.3 nm (Figure 3a).This increase marks the transition between a tip oscillation dominated by attractive forces to a tip oscillation dominated by repulsive forces. The increase in the amplitude curve is also reflected in the phase shift curve (Figure 3b) where the phase shift changes from about 110° to 65°. The initial values of the deflection, position and velocity determine the *z_c_* value where the jump occurs. The inset (Figure 3a) shows the coexistence of two amplitude values for the same *z_c_*. This coexistence generates a hysteresis loop [11]. We note that in the attractive regime, the phase shift increases from the non-interacting value (90°) with decreasing *z*_c_. However, in the repulsive regime, 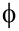 decreases with decreasing *z*_c_.

#### Low versus high *Q* values

The above considerations apply for environments with relatively high *Q* values (10–500), which experimentally usually implies air environments. For simulations performed in liquid, *Q ≈* 1–5, and the tip motion is markedly different [20,22–24,58]. First, the AFM operation is controlled by repulsive forces because the attractive forces are highly screened in liquid. The absence of an attractive regime in liquid implies that the phase shift decreases by decreasing *z*_c_. Second, for the same instrument, the noise in the amplitude is higher in liquid. This happens because the noise is proportional to 1/(*dA*/*dz*_c_) [47]. The slope of the amplitude curve in liquid is 0.7 while in air for the same system it is 1 (Figure 3a). Third, in liquid the observed cantilever motion depends on the microcantilever excitation method [22,58]. The code is written to simulate the dynamics of directly excited microcantilevers (photo-thermal or magnetic excitation).

The amplitude spectrum also depends on the medium (*z*_c_ = 5 nm). The anharmonicity of the motion is higher in liquid [20,59]. For all the frequencies except the fundamental, the amplitude of the harmonics is higher in water (Figure 3c).

#### Imaging soft and hard materials

**Figure 4:**
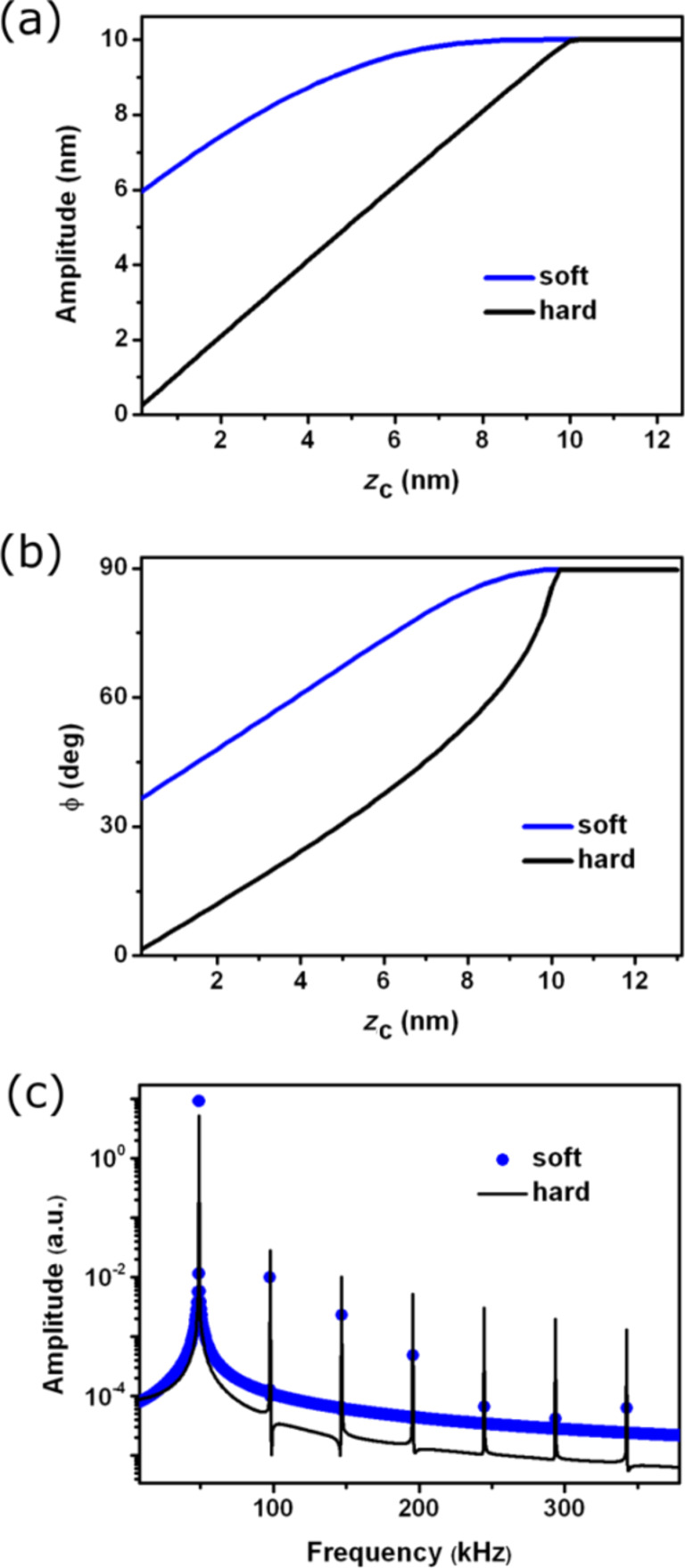
AM-AFM comparison of cantilever dynamics, hard versus soft materials. (a) Amplitude versus average tip–surface distance curves for hard and soft surfaces. (b) Corresponding phase shift curves. (c) Spectra of the amplitude components taken at *z*_c_ = 5 nm.

The cantilever dynamics in AM-AFM shows some subtle differences depending on the effective Young’s modulus of the interaction. Figure 4 shows the amplitude, phase shift and harmonic components for two materials characterized by an *E*_s_ of 50 MPa and 50 GPa. The amplitude curve shows a significant difference in the slope. In the stiffer material the slope is nearly 1 while for the softer material the slope is about 0.7. Because the noise in the amplitude depends on the slope as 1/slope, the smaller value of the slope implies that in softer materials it is more difficult to achieve high resolution. The phase shift (Figure 4b) decreases more rapidly with the average distance in the stiffer material. The amplitude spectrum shows that strength of the higher harmonics also depends on the material. Higher components are observed in the stiffer material (Figure 4c).

#### Viscoelastic materials

**Figure 5:**
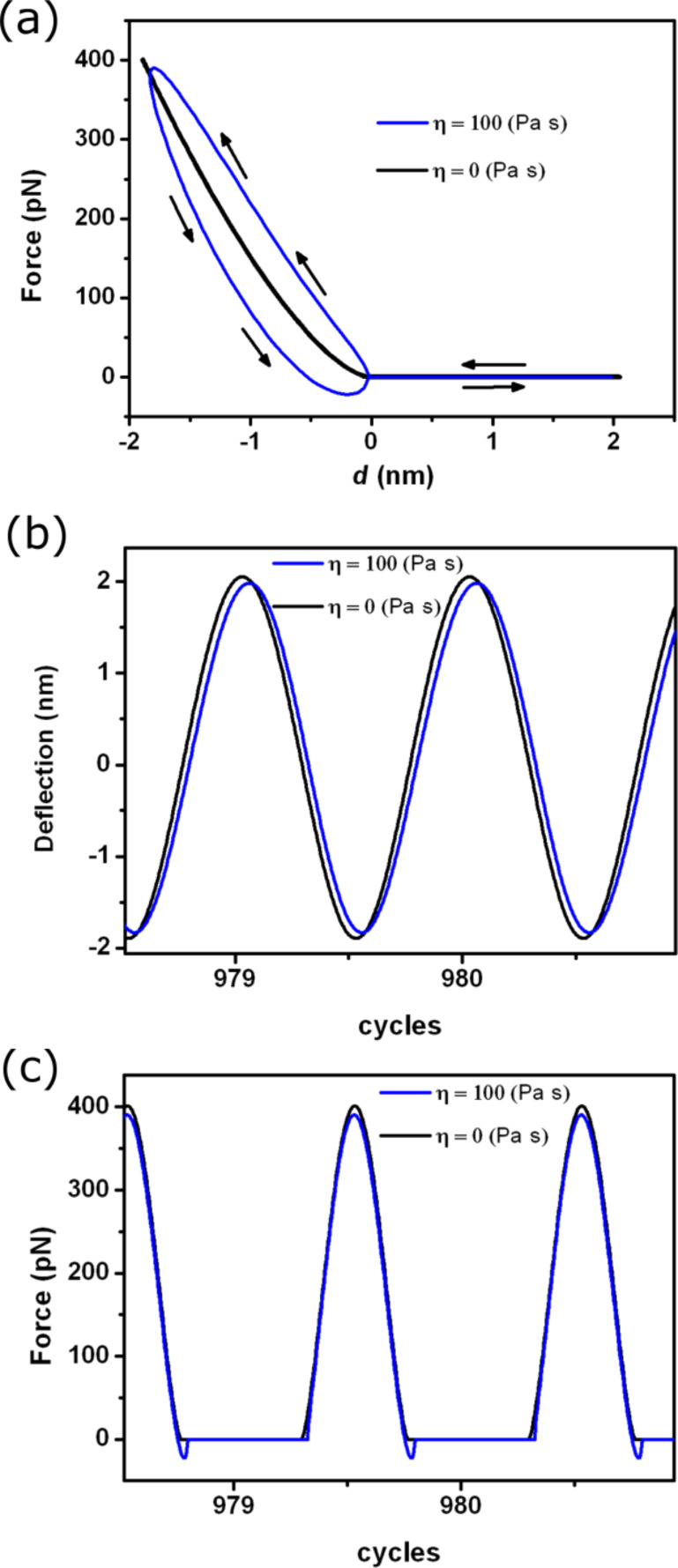
AM-AFM simulations for a viscoelastic material. (a) Force–distance curve for the linear viscous model. The blue curve represents the conservative force given by the Hertz model. (b) Tip deflection as a function of time for an elastic and a viscoelastic material. (c) Instantaneous force at *z*_c_ = 0.2 nm.

Experiments involving soft matter usually imply the existence of a viscoelastic response. Figure 5a shows the viscoelastic force calculated with the Garcia–San Paulo expression (Equation 20). Far from the sample surface, the force is zero (not including long-range forces) and upon contact, the repulsive force increases until a maximum is reached. During retraction, an attractive force appears due to the viscoelastic response of the material. Then the force depends on the direction the tip motion. The thick blue line shows the elastic component of the force.

The tip deflection is shown in Figure 5b. The amplitude is slightly smaller in presence of viscous material and the oscillation is phase shifted with respect to the absence of a viscoelastic response. The instantaneous force shows a region of attractive force when the tip withdraws from the sample surface (Figure 5c). This effective attractive force originates from the viscoelastic interactions.

We have also compared dForce and VEDA simulations [41–42] for several AM-AFM cases. Both simulators give similar results with some minor numerical differences. The compatibility of dForce with different operating systems (Windows, Mac and Linux), the autonomy of running dForce without internet access, and the flexibility by offering customizable plots and interactions forces, highlight several advantages dForce offers.

#### Bimodal AM

The simulations were performed for the bimodal AM configuration where the first two flexural modes are excited and an amplitude modulation feedback controls the amplitude of the first mode [33]. The parameters used in the bimodal AM simulations are presented in Table 4.

**Table 4:** Summary of the parameters used in the numerical simulations of bimodal AFM described in Figure 6 and Figure 7.

Figure	*R* (nm)	*f*_1_ (kHz)	*k*_1_ (Nm)	*Q*_1_	*A*_01_ (nm)	*f*_2_ (kHz)	*k*_2_ (Nm)	*Q*_2_	*A*_02_ (nm)	*E*_s_ (Pa)	*H* (J)

6	1	50	0.9	10	10	310	35.2	62.7	1	1 G	4 × 10^−20^
7	20	48.9	0.9	255	17	306.6	35.2	1000	1	1 G	4 × 10^−20^ 9 × 10^−20^

**Figure 6:**
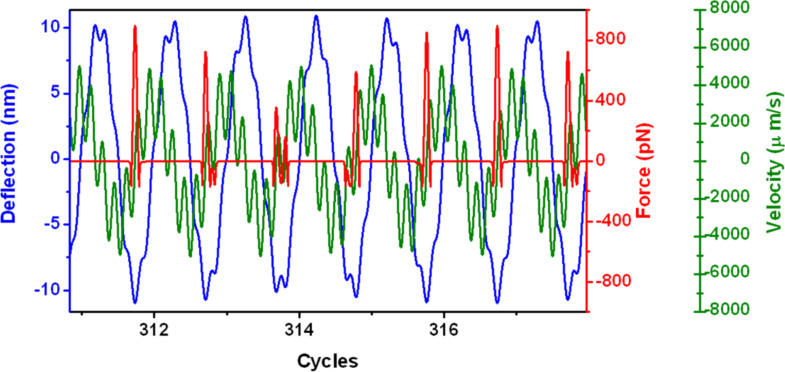
Bimodal AFM tip motion. The tip oscillation (blue), instantaneous force (red) and velocity (green) are shown. Notice that in this particular case the period of the oscillation is 6 times the period of the first mode. Data obtained at *z*_c_ = 9 nm.

Figure 6 shows the tip response under the influence of excitation and the tip–surface interactions. The periodicity of the signal occurs at a frequency that is a multiple of both the first and the second modes. The instantaneous force shows the region of attractive and repulsive forces and the variation of the peak forces over different periods of the first mode.

**Figure 7:**
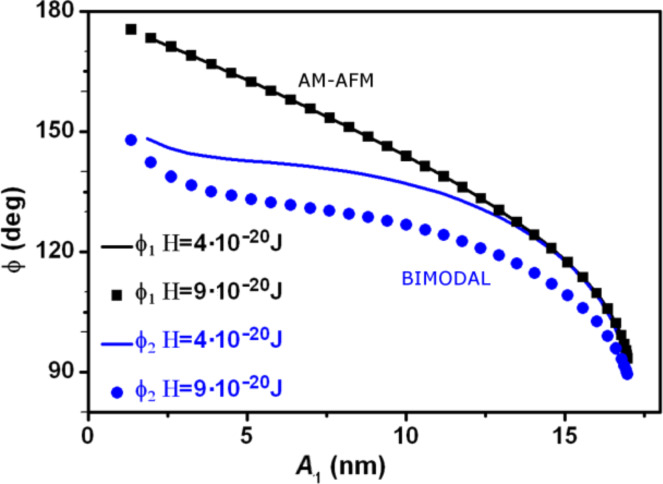
Material contrast in bimodal AFM. Phase shift as a function of the set-point amplitude in bimodal AFM (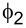 versus *A*_1_). To illustrate the bimodal effect, we show the simulations for regular AM-AFM (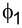 versus *A*_1_).

In bimodal AM, the phase shift of the second mode is the observable used in heterogeneous samples to separate regions of different material properties. Figure 7 shows the dependence of the phase shift as a function of the set-point amplitude and the material properties (changes in the Hamaker constant). The phase shift 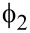 (attractive regime) has a sudden increase that it is followed by a region where the 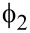 seems to saturate but then suddenly increases for small *z_c_* values. The shape of the curve is reproduced for other materials (*H* values).

To illustrate the differences with the AM-AFM operation we also plot the phase shift for the same values of *H* (in this case 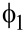). The phase shift in AM-AFM is not sensitive to changes in the conservative terms of the interaction. We remark that the above result holds for a system without dissipative elements [5].

## Conclusion

We have developed an interactive simulation environment, based on open source code, to simulate the full cantilever dynamics in both amplitude modulation and bimodal AM force microscopies. The code is both robust and numerically accurate. It incorporates the most relevant interaction force models that apply for dynamic AFM experiments in air and liquid. The simulator has been tested over the years on a wide variety of different AFM conditions.

The simulator will help to clarify and understand any arising complexity in the tip motion found in both amplitude modulation and bimodal AFM and, in the process, to establish the relationship between material properties, forces and observables for a given experiment. Because this is open source software, the advanced user could incorporate additional libraries as desired. Finally, the use of dForce must be accompanied by an understanding of the physics behind the simulations in order to select appropriate input parameters that will generate meaningful and correct results.
